# Consumer Choices in the Functional Food Market: A Review of Determinants of Purchasing Behavior

**DOI:** 10.3390/foods15081319

**Published:** 2026-04-10

**Authors:** Jagoda Żurek, Mariusz Rudy, Dariusz Dziki

**Affiliations:** 1 Department of Financial Markets and Consumer Finance, Faculty of Economics and Finance, University of Rzeszow, Ćwiklińskiej 2, 35-601 Rzeszow, Poland; jzurek@ur.edu.pl; 2 Department of Agricultural Processing and Commodity Science, Institute of Food and Nutrition Technology, Faculty of Technology and Life Sciences, University of Rzeszow, Zelwerowicza 4, 35-601 Rzeszow, Poland; 3 Department of Thermal Technology, University of Life Sciences in Lublin, Głęboka 31, 20-612 Lublin, Poland

**Keywords:** functional food, consumer behavior, purchase determinants, consumer attitudes, sociodemographic factors, food purchasing decisions, food choice

## Abstract

The article provides a comprehensive review of empirical studies on consumer attitudes, motivations, and behaviors in the functional food market. The main objective of this study is to identify groups of determinants and to update and systematize current knowledge on the influence of various factors on consumer purchasing decisions in this market. Based on an analysis of international research published between 2004 and 2025, four key groups of determinants were identified: (1) health- and trust-related factors, (2) cognitive and psychological factors, (3) perceptual and product-related factors, and (4) socio-demographic and segmentation factors. The analysis confirms that purchasing decisions in this product category are complex and multidimensional. They result from the interaction between rational factors (health-related and cognitive) and emotional-symbolic factors (psychological and sensory). The strongest predictors of functional food acceptance include perceived health benefits, trust in producers and information sources, sensory attractiveness, and product naturalness. Socio-demographic characteristics, such as age, education level, and income, further differentiate purchasing intentions and behaviors. Overall, the findings highlight the need for further comparative and cross-cultural research, as cultural and economic conditions may significantly shape consumer decisions across markets. The results obtained have both theoretical and practical implications. They contribute to a better understanding of consumer decision-making processes and emphasize the importance of promoting health awareness.

## 1. Introduction

In recent years, the global food market has undergone substantial transformations driven by growing consumer awareness of the relationship between dietary patterns and health outcomes [1]. At the same time, demographic changes, rising education levels, and increasing interest in preventive health have encouraged consumers to seek products with scientifically proven health benefits [2,3]. In this context, functional foods have gained particular importance. These products provide not only basic nutrients but also additional health benefits that support the body and reduce the risk of chronic diseases [4,5,6]. This segment has become one of the fastest-growing areas of the food market. It responds to the needs of consumers who aim to improve their quality of life through more conscious dietary choices [7,8,9].

The concept of functional foods originated in Japan. As early as the 1980s, initiatives were introduced to improve public health and reduce healthcare costs. In 1988, Otsuka Pharmaceutical introduced the first product of this type—a non-alcoholic beverage enriched with dietary fiber [8]. At the same time, the Ministry of Health and Welfare developed the Foods for Specified Health Use (FOSHU) system. This system includes products with scientifically proven health effects, such as supporting gut microbiota, regulating nutrient absorption, and reducing the risk of metabolic syndrome. FOSHU foods are classified into several categories related, among others, to digestive, bone, and cardiovascular health, as well as cholesterol and blood glucose reduction [10,11].

In Europe, the concept of functional food was further developed within the Functional Food Science in Europe (FUFOSE) project coordinated by the European Commission. According to the definition adopted in this initiative, “a food can be regarded as ‘functional’ if it is satisfactorily demonstrated to affect beneficially one or more target functions in the body, beyond adequate nutritional effects, in a way that is relevant to either an improved state of health and well-being and/or reduction of risk of disease” [12] (p. S6). This definition emphasizes that functional foods remain conventional foods rather than supplements or medicines. Their health effects must be scientifically proven and achievable within a normal daily diet. FUFOSE also proposed distinguishing between two types of health claims:

– Type A—Enhanced Function Claims, referring to the beneficial effects of nutritional and non-nutritional components on psychological, physiological, or biological functions of the body,
– Type B—Reduction of Disease Risk Claims, related to reducing the risk of disease occurrence [12].

Initially, functional food was defined as products that improve health by modulating physiological functions [13]. Currently, they are understood more broadly as natural or modified food products enhanced through technological or biotechnological processes. Their beneficial effects should occur at intake levels consistent with typical dietary consumption [14].

Today, the European Food Safety Authority (EFSA) does not define functional food as a separate category. Instead, it focuses on evaluating health claims related to specific ingredients and their physiological effects. The legal framework is provided by Regulation (EC) No 1924/2006 of the European Parliament and of the Council of 20 December 2006 on nutrition and health claims made on foods [15]. Under this regulation, two main types of claims are distinguished in the European Union: nutrition claims and health claims [16]. Health claims are further divided into functional health claims, relating to the physiological effects of food components; disease risk–reduction claims, indicating the potential to reduce the likelihood of disease; and claims referring to children’s growth and development [15,16].

A more recent definition has been proposed by the Functional Food Center (FFC, USA). According to this approach, functional foods are defined as “natural or processed foods that contain biologically-active compounds; which, in defined, effective, non-toxic amounts, provide a clinically proven and documented health benefit utilizing specific biomarkers, to promote optimal health and reduce the risk of chronic/viral diseases and manage their symptoms” [17,18] (p. 214; p. 95). According to Gur et al. [19] and Martirosyan and Ekblad [18], the definition of functional food includes several key elements describing both composition and mechanisms of action. It refers to natural or processed food products containing biologically active components that exert a measurable, beneficial effect on human health. These components must be present in precisely defined, effective, and safe quantities that ensure a physiological response without toxicity risk. Importantly, the health-promoting properties of such products should be scientifically verified—preferably through clinical studies and biomarker analyses demonstrating actual changes in biological functions or disease risk. In this perspective, functional foods aim to support prevention, mitigation, or control of chronic diseases and their symptoms through regular inclusion in the daily diet [18,19].

From a public health perspective, functional foods represent a promising strategy for reducing non-communicable diseases. They may contribute to improving population health through better dietary patterns [20]. According to Sgroi et al. [8], citing Euromonitor data, global average spending on functional foods amounts to 26 euros per person, although this varies across regions. Asia is the largest market, with Japanese consumers spending approximately 150 euros annually. In contrast, the United States and Canada account for about 25% of the global market, while Europe remains more fragmented [8].

As the popularity of functional foods increases, research on consumer decision-making becomes increasingly important. Consumer choices are influenced not only by health awareness, but also by perceived effectiveness, trust, knowledge, price, and availability [3,21,22,23,24,25]. Moreover, psychological and social factors such as the need for health control, social influence, and lifestyle also play a significant role [26,27]. Understanding these determinants helps explain why consumers choose such products and whether declared health benefits translate into actual behavior.

Special attention should be given to identifying groups of determinants that influence purchasing decisions, including health and trust-related factors, cognitive and psychological factors, perceptual and product-related factors, as well as socio-demographic and segmentation factors.

The analysis of consumer purchasing behavior requires a solid theoretical foundation that explains both rational and psychosocial decision-making mechanisms. In this context, the Theory of Planned Behavior (TPB) is particularly useful, as it assumes that purchase intentions are shaped by attitudes toward the product, social norms, and perceived behavioral control [28]. In addition, the Health Belief Model (HBM) suggests that consumer behavior is influenced by health motivation, perceived threat (including perceived susceptibility and severity), and the evaluation of expected benefits in relation to perceived barriers and costs [29]. Another important perspective is the concept of Customer Perceived Value, which indicates that purchasing decisions result from a subjective evaluation of the relationship between perceived benefits and costs [30]. In the context of functional foods, this means that consumer choices are driven not only by health benefits but also by factors such as taste, naturalness, price, trust, and overall product attractiveness.

The purpose of this work is to provide a overview of how these groups of determinants influence the acceptance, purchase intention, and actual choices of consumers across different market contexts. It also integrates international research findings to identify both common patterns and culturally driven differences in consumer behavior.

It is assumed that consumer behavior toward functional food is multidimensional and results from the interaction of these mechanisms. Based on this assumption, the analysis is structured around four groups of determinants: (1) health- and trust-related factors, (2) cognitive and psychological factors, (3) perceptual and product-related factors, and (4) socio-demographic and segmentation factors.

The structure of the article is as follows. Section 2 presents the methodology of the literature review, including the search strategy, selection criteria, and study selection process. Section 3 provides an overview of the functional food market and its development conditions. Section 4 discusses the main groups of determinants of consumer purchasing behavior in the functional food market. Section 5 presents the theoretical implications, practical implications, study limitations, and directions for future research. Finally, Section 6 provides the conclusions of the review.

## 2. Methodology of the Review

This study provides a comprehensive integration and synthesis of findings from previously published research on consumer behavior toward functional foods. It adopts an integrative review approach, enabling a comprehensive synthesis of evidence derived from diverse empirical studies as well as review papers published in peer-reviewed scientific journals [31]. The study follows an integrative review methodology, while elements of the PRISMA framework were applied to structure the identification, screening, and selection of studies.

The authors synthesized and interpreted primary research results concerning consumer attitudes, motivations, and behaviors toward functional foods, while review articles were included to support the conceptual background and contextualize the findings. Such an approach enables the integration of evidence from diverse sources and supports the comparison of conclusions obtained across different cultural and methodological contexts. The advantage of this type of review lies in its ability to enhance the precision and validity of interpretations by juxtaposing findings from multiple studies, allowing the identification of recurring trends and key determinants across various consumer groups.

The following research questions were formulated to guide the analysis:

- RQ1: What groups of factors determine consumer purchasing decisions in the functional food market?
- RQ2: How do the identified determinants influence consumer attitudes, purchase intentions, and actual purchasing behavior?
- RQ3: What theoretical, practical, and research implications emerge from the current state of knowledge on consumer behavior toward functional foods?

The temporal scope of the review includes publications from 2004 to 2025, while earlier studies were incorporated only to clarify the definitional and conceptual background. This period was selected due to the dynamic development of the functional food market and the growing number of empirical investigations examining consumer behavior since the early 2000s.

To identify relevant publications, two major scientific databases were used: Scopus and Web of Science. The search strategy was based on combinations of keywords related to functional foods and consumer behavior. In particular, the following terms were used: “functional food” OR “functional foods”, “consumer behavior”, “consumer attitudes”, “purchase intention”, “consumer acceptance”, “health claims”, “perceived health benefits”, “trust”, “sensory attributes”, “taste”, “product attributes”, “willingness to pay”, and “food neophobia”.

These keywords were combined using Boolean operators (AND, OR), with their structure adapted to the specific search requirements of each database. The literature search was conducted in May 2025. Scopus (Elsevier, Amsterdam, The Netherlands), considered the largest source of bibliometric data worldwide, comprises over 100 million records, including approximately 25.2 million open-access documents [32,33]. Web of Science (Clarivate Analytics, London, UK) provides access to over 34,000 peer-reviewed scientific journals and includes an archive dating back to 1864 [34].

**Figure 1:**
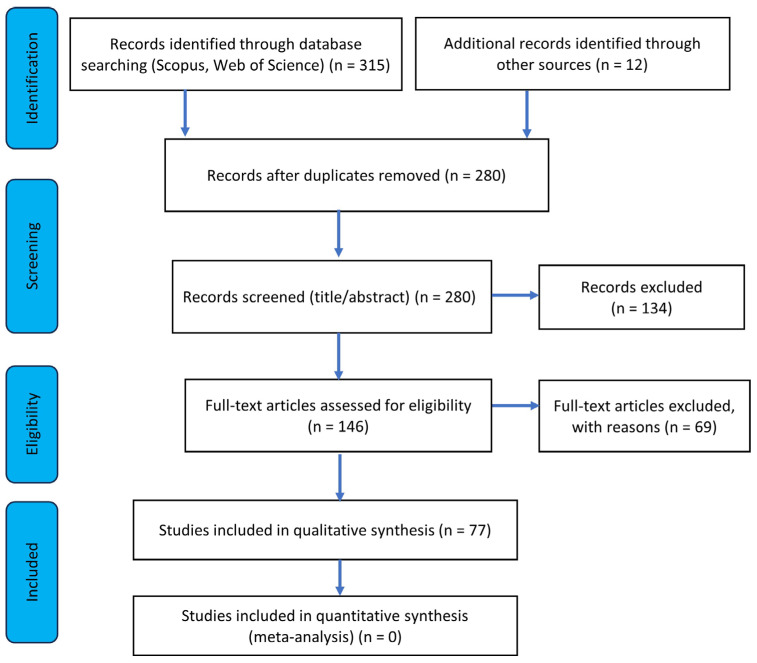
PRISMA flow diagram of the literature selection process. Source: authors’ own elaboration based on PRISMA [35].

The study selection process was conducted in accordance with PRISMA guidelines [35] and consisted of several stages. This approach ensures methodological transparency while preserving the flexibility and inclusiveness characteristic of integrative reviews. Additionally, a manual search of the reference lists of selected articles was conducted to identify potentially relevant studies not captured in the database search. The initial search yielded a total of 315 publications. After removing duplicates, 280 records remained and were screened based on titles and abstracts. As a result, 146 articles were selected for full-text assessment. Following the application of inclusion and exclusion criteria, 77 publications were included in the final analysis. The review covered empirical studies (both quantitative and qualitative) as well as review papers focusing on consumer behavior toward functional foods. The study selection process is presented in Figure 1.

Exclusion criteria included: publications without full-text availability, studies published before 2000, opinion papers, reports, conference abstracts, materials lacking empirical data, doctoral theses, editorial articles, popular science papers, and research not published in English. The final literature corpus forms the basis for identifying the four main groups of determinants shaping consumer behavior in the functional food market.

## 3. Functional Food Market Overview

Natural functional foods include unprocessed or minimally processed products such as vegetables, fruits, nuts, whole grains, legumes, and fatty marine fish. These products are valuable sources of nutrients, fatty acids, dietary fiber, antioxidants, and other bioactive compounds that support the proper functioning of the human body [7,36,37,38,39,40,41]. In contrast, technologically processed functional food are developed through deliberate modifications of their composition. These modifications include enrichment with unsaturated fatty acids (e.g., omega-3), vitamins, and minerals, as well as reductions in fat, lactose, or cholesterol content. Such interventions aim to increase the concentration of bioactive components and enhance the health-promoting properties of these products [1,39,42,43,44,45]. Table 1 presents selected bioactive components found in foods and their potential health effects.

The literature identifies four fundamental criteria that define functional foods: scientifically documented health benefits, the nature of the product, expanded nutritional functionality, and the feasibility of regular incorporation into the diet [18,19]. A summary of these criteria is presented in Table 2.

In the context of functional food production, innovative technologies that enable the preservation of nutritional value and product stability are gaining increasing importance. One such modern solution is nanotechnology, which utilizes nanoscale structures (up to 1000 nm) that, due to their large surface area and unique physicochemical properties, allow for the development of foods with improved characteristics—including enhanced bioavailability of bioactive compounds, extended shelf life, and better protection of oxygen- and heat-sensitive components [82,83,84,85]. Incorporating nanoparticles into the structure of functional products increases the resistance of bioactive components to unfavorable conditions in the human gastrointestinal tract, such as exposure to digestive enzymes, pH fluctuations, and oxygen. This enhances their stability and enables more effective release and absorption in specific sections of the digestive system, resulting in higher bioavailability and biological effectiveness of these compounds [85,86,87].

One of the most promising technological innovations in the food industry is 3D food printing [88]. This technique allows for the creation of products layer by layer using so-called “food inks” characterized by specific rheological properties. Such an approach makes it possible to design foods with personalized nutritional composition, texture, and flavor tailored to individual consumer needs. Moreover, 3D printing opens new opportunities for functional foods by enabling the enrichment of products with vitamins, minerals, and bioactive compounds (e.g., polyphenols) and the development of snacks targeted at specific age groups or health-related needs [89]. An example of such an innovative application is the development of a functional snack produced from natural bioactive ingredients such as mandarin peel, kiwifruit, and pectin, which served as raw material for 3D printing. The resulting product exhibited a high content of phenolic compounds and demonstrated anti-inflammatory and antioxidant properties [90], confirming the potential of 3D printing in creating next-generation functional foods.

Although fermentation is one of the oldest food-processing methods, its modern application in functional food production has taken on an innovative character. By using selected strains of microorganisms and advanced biotechnological techniques, it is now possible to control fermentation processes in ways that increase bioactive compound content, improve nutrient bioavailability, and support a beneficial gut microbiome. Products such as kefir, kimchi, miso, and fermented dairy beverages exemplify foods that combine traditional techniques with contemporary approaches to health promotion. In this sense, fermentation is no longer merely a preservation method but also a tool for designing foods with specific health-enhancing functions [91].

The complex nature of the functional food market means that its future growth largely depends on consumer attitudes and purchasing decisions. Understanding the factors that shape these choices is therefore essential for the effective development of this segment. Growing consumer interest in foods bearing health claims—indicating scientifically verified health-promoting properties beyond basic nutritional functions—points to increasing awareness of the role of diet in maintaining health. This trend may contribute to improved quality of life and reductions in morbidity and mortality [92].

Given the growing influence of consumers in shaping the functional food market, identifying the determinants of purchasing behavior in this area becomes increasingly important. Analyzing consumers’ motivations, barriers, and decision-making processes provides deeper insight into the mechanisms underlying the acceptance and selection of functional products, as well as their role within contemporary consumer behavior patterns.

## 4. Determinants of Consumer Purchasing Behavior

The process of consumer purchasing decision-making in the functional food market is complex and influenced by numerous factors that shape preferences, attitudes, and purchase intentions. Based on the literature, four main groups of determinants affecting purchasing behavior have been identified: (1) health- and trust-related factors, (2) cognitive and psychological factors, (3) perceptual and product-related factors, and (4) socio-demographic and segmentation factors. The adopted classification has an analytical character and is intended to organize the diverse factors identified in the literature. However, these categories are not entirely distinct. The individual groups of determinants were distinguished based on the dominant dimension of their influence on consumer decisions. Health- and trust-related determinants primarily refer to perceived health benefits and the credibility of information and market actors. In contrast, cognitive and psychological determinants include information processing mechanisms, beliefs, motivations, and social norms that shape attitudes and purchase intentions. Perceptual and product-related determinants focus on the evaluation of specific product attributes, such as taste, naturalness, and price, while socio-demographic and segmentation factors play a moderating role by influencing the strength and direction of the remaining determinants. It should be noted, however, that the boundaries between these categories are not strictly defined. For example, health perception and trust may also have a psychological dimension. Therefore, the adopted approach is heuristic in nature and aims to enhance the clarity of the analysis and facilitate the synthesis of research findings. Each of these categories reflects a distinct dimension of consumer decision-making—from health- and trust-related factors, through cognitive and psychological aspects, to product characteristics and socio-demographic influences.

**Figure 2:**
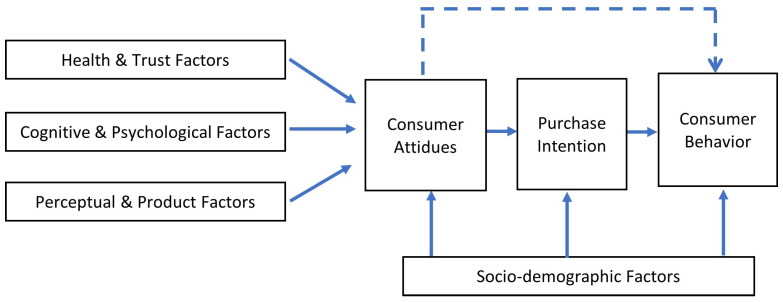
Conceptual model of the determinants of consumer purchasing behavior in the functional food market. Source: authors’ own elaboration.

The adopted approach is illustrated by a conceptual model (Figure 2), which presents the relationships between the identified groups of determinants, consumer attitudes, purchase intention, and purchasing behavior. The arrows indicate the relationships between the variables presented in the model.

The following part of this section presents a review of the literature and empirical studies illustrating the influence of these determinants on consumer attitudes and behaviors toward functional foods. A synthetic summary of the key findings from international studies is provided in Table 3.

### 4.1. Health- and Trust-Related Determinants

Research consistently indicates that perceived health benefits and trust in information sources and market actors form the foundation of consumer acceptance of functional foods. Across studies, consumers are more likely to choose functional products when they perceive them as tools for prevention or alleviation of specific health concerns and when health-related claims are regarded as credible.

Experiences associated with the COVID-19 pandemic strengthened consumers’ health orientation. Interest in prevention and immunity increased, which translated into higher demand for products with scientifically confirmed health-promoting properties [28,106]. As a result, functional foods gained importance as a category that integrates nutritional and health-related dimensions and are increasingly perceived not only as part of the diet but also as tools supporting overall health and well-being [21].

Siegrist, Shi, Giusto, and Hartmann [93] analyzed Chinese consumers’ attitudes toward functional foods, focusing on their willingness to purchase products with declared health benefits. The results showed that most respondents (approximately 65%) preferred functional foods over conventional alternatives. The authors emphasized that consumers in China display strong interest in products offering clearly defined health benefits, with health motivation being a major determinant of purchasing decisions. Individuals who considered health an important criterion in food choice were more likely to express willingness to purchase functional products [93].

Within the group of health-related determinants, concerns related to physical mobility (e.g., bones, joints, muscles) are particularly significant. Mirosa and Mangan-Walker [21] demonstrated that Chinese consumers highly value the maintenance of physical mobility and expect its importance to increase with age. Moreover, stronger concerns about mobility-related diseases increase the likelihood of purchasing functional foods for preventive purposes.

In the study by Rasanjalee and Samarasinghe [94], conducted among 280 consumers in Sri Lanka, the influence of knowledge, perceived necessity, safety, trust, and perceived rewards on attitudes toward functional foods was examined. The results revealed that while knowledge, perceived necessity, and safety negatively affected attitudes, trust and perceived health rewards emerged as the strongest positive determinants of acceptance. Consumers who perceived clear and tangible health benefits exhibited the most favorable attitudes toward functional foods. These findings confirm that purchasing decisions are driven more strongly by emotional conviction about benefits than by factual knowledge alone.

Szakos et al. [3] showed that the importance of functional foods varies by age and consumers’ health conditions: older individuals more often reported health issues, but this did not always translate into greater acceptance of functional foods; an exception was migraine, more commonly reported among younger groups. Statistically significant differences were observed for cardiovascular diseases, arthritis, allergies, skin problems, and lactose intolerance.

Urala and Lähteenmäki [22] identified seven dimensions of attitudes toward functional foods, including perceived health rewards, trust, perceived necessity, nutritional risk, and the relationship between taste and healthiness. The strongest predictor of consumption willingness was the subjective sense of health reward, which is consistent with findings from other studies, including those conducted in Sri Lanka [94].

Plasek et al. [105] showed that consumers particularly value functional foods in the context of digestive issues, reduced immunity, and high cholesterol. Higher education was associated with choosing immunity-related products, while age above 36 was linked to preferences for digestive and cardiovascular prevention. In Siegrist et al. [93], health motivation and trust in the food industry positively influenced purchase intention, with clear cultural differences, including higher willingness to purchase in China than in Germany [93].

Huang et al. [23] confirmed that trust in the food system and health consciousness positively influence attitudes and purchase intentions. High price could weaken purchase willingness, but this effect diminished among consumers with high health awareness. Food neophobia moderated these relationships—at lower levels of neophobia, price was more often perceived as a signal of quality [23].

Health-related determinants and trust remain key demand drivers. The analysis of the reviewed studies indicates a high level of consistency regarding the importance of health motivation and trust as key determinants of purchasing behavior. However, important differences emerge with respect to the role of knowledge and risk perception. In some studies, nutritional knowledge enhances the acceptance of functional foods, while in others it leads to greater skepticism toward health claims, which may result from a more critical evaluation of the credibility of available information. The findings also suggest that the importance of health- and trust-related determinants is strongly dependent on cultural context and the level of consumer health awareness. These differences may stem not only from varying cultural norms but also from regulatory environments and dietary traditions, which influence how health claims are interpreted, the level of trust in products, and perceptions of their naturalness.

International research further indicates that the level of acceptance of functional foods varies significantly across regions. Studies conducted in Asian countries report higher willingness to purchase and greater openness toward products with health-related claims, which is associated with stronger health orientation and higher trust in the functional aspects of food [93]. In contrast, in many European countries greater importance is attached to product naturalness, authenticity, and the credibility of health-related communication, which may reflect different dietary traditions and a more critical approach to health claims [22,96]. Similar patterns can also be observed at the global level. For example, Japan—one of the most developed markets for functional foods—exhibits a high level of consumer acceptance, which is linked to the long-standing development of this category and strong regulatory support [107].

### 4.2. Cognitive and Psychological Determinants

Consumer behavior toward functional foods is shaped not only by nutrition knowledge and health-related beliefs but also by psychological factors such as emotions, the need for control, social norms, and symbolic motivations. The literature increasingly emphasizes that purchasing decisions in this category are not purely rational but also reflect self-image, social influences, and emotional needs.

The study by Barauskaite et al. [27], conducted among 900 Lithuanian consumers, confirmed the importance of socio-psychological factors in shaping attitudes toward functional foods. Demonstrative consumption (the desire to signal a healthy lifestyle) and susceptibility to social norms positively affected the perceived attractiveness and distinctiveness of functional products. In contrast, self-control motivation had the opposite effect—individuals guided by a strong need to control their diet showed lower openness to novelty. These findings suggest that choosing functional foods may serve a signaling function, reflecting consumers’ health identity and social status.

Nystrand and Olsen [95] applied an extended version of the Theory of Planned Behavior, incorporating self-efficacy, descriptive and injunctive norms, and hedonic and utilitarian values. In a sample of 810 Norwegian consumers, self-efficacy—the belief that one is capable of regularly consuming such products—emerged as the strongest predictor of intention to consume functional foods. Both descriptive norms (derived from observing others’ behaviors) and injunctive norms (social expectations) significantly strengthened intention, with descriptive norms showing a stronger effect. Utilitarian values (linked to functional health benefits) positively shaped attitudes, whereas hedonic values (pleasure, taste) had a negative influence. The authors emphasized that increasing market acceptance requires improving the “hedonic value” of functional foods, that is, combining health benefits with pleasurable consumption experiences.

These findings indicate that consumer decisions are largely shaped by psychological and social mechanisms, the importance of which may vary depending on cultural context. Cross-cultural research suggests that in societies with a higher level of collectivism, social norms and the influence of the surrounding environment play a more significant role in purchasing decisions [108]. A study conducted in Norway and Portugal showed that collectivism positively influenced subjective norms, attitudes, perceived price, and environmental concern in relation to organic food, and these factors, in turn, significantly shaped purchase intention. At the same time, some relationships were observed only in specific samples—for example, the positive association between collectivism and perceived product availability was statistically significant only in Portugal [109]. Importantly, collectivism did not affect the level of consumers’ health awareness, which proved to be one of the strongest predictors of purchase intention. This suggests that health-related factors may influence consumer decisions relatively independently of cultural context, whereas social and normative factors appear to be more sensitive to cultural differences.

In the study by Verbeke [96], conducted among Belgian consumers (*n* = 215), belief in health benefits was the strongest positive determinant of acceptance of functional foods. The presence of a chronically ill family member also contributed to more positive attitudes, whereas a high level of self-reported knowledge about functional foods was associated with lower acceptance—possibly reflecting greater skepticism among better-informed consumers. This effect weakened with age, suggesting that older consumers adopt a more pragmatic approach to health-oriented products [96]. These findings indicate that acceptance depends not only on the level of knowledge but also on how consumers interpret that knowledge—through the lens of trust and personal beliefs. Cognitive and psychological determinants reveal that attitudes toward functional foods result from a complex interaction between knowledge, emotions, and social influence. The findings are partly consistent in highlighting the importance of health beliefs, social norms, and self-efficacy as key determinants of purchase intention. At the same time, important discrepancies emerge regarding the role of knowledge. While in some studies it enhances the acceptance of functional foods, in others it leads to greater skepticism toward health claims. This may result from a more critical evaluation of information credibility among better-informed consumers. Furthermore, the results suggest that emotional and social factors—such as identification with a particular lifestyle and the influence of social norms—often strengthen purchasing decisions regardless of the level of knowledge. This implies that the market success of functional foods depends not only on consumer education but also on the effective integration of health-related communication with elements that build trust and create positive consumption experiences.

### 4.3. Perceptual and Product-Related Determinants

Product attributes constitute one of the key groups of determinants influencing consumer purchasing decisions in the functional food market. Characteristics such as taste, composition, format, presentation, and price shape not only the perception of quality but also the level of trust and acceptance. Unlike conventional products, functional foods additionally require credibility of the declared health benefits, making sensory and informational perception particularly important. Within this group of determinants, the following elements can be distinguished: carrier and ingredient congruence, sensory attributes, labeling and packaging, naturalness/transparency, and price as well as willingness to pay (WTP).

One of the most important factors determining the acceptance of functional foods is the type of base product to which bioactive substances are added. The so-called product carrier provides the sensory and technological foundation, and its nature (e.g., yogurt, juice, bread, dairy beverage) largely determines the perceived credibility and health-related attractiveness of the product [99,110,111]. Studies by Temesi et al. [97] and Huang et al. [98] indicate that consumers show higher acceptance when the carrier and functional ingredient are perceived as congruent in terms of health function—for example, probiotic yogurt or vitamin C–enriched juice.

Temesi et al. [97] found that Hungarian consumers’ acceptance of functional foods was primarily determined by sensory and cognitive factors. Expected taste was the strongest determinant, increasing the likelihood of positive evaluation more than threefold. In addition, congruence between the health effect of the carrier and the added ingredient increased the probability of acceptance by approximately 30%. These results confirm that sensory satisfaction remains an essential condition for the success of functional products. Similarly, in a study by Williams et al. [99] in Australia, sensory attractiveness and carrier type exerted a stronger influence on purchasing intention than the content of the health claim itself, highlighting the dominant role of product form over messaging. In turn, Huang et al. [98] demonstrated that among Chinese consumers, product attributes and trust in information sources were the key determinants of purchasing intention, with the product carrier being a stronger predictor than the declared health benefits [98]. A synthesis of these findings highlights a clear trade-off between perceived health benefits and sensory attributes. Although consumers often declare the importance of health aspects, taste and consumption pleasure frequently act as necessary conditions for product acceptance. Evidence suggests that even a high level of perceived health benefits does not compensate for poor sensory quality, indicating a fundamental tension between health value and hedonic attributes. At the same time, some studies indicate a reciprocal relationship, where positive taste perception can enhance the perceived health value of a product. This interplay suggests that the market success of functional foods depends on the ability to balance health-related benefits with sensory appeal.

Cavaliere et al. [112], although analyzing products with nutrition and health claims rather than strictly functional foods, demonstrated that the way health benefits are communicated significantly influences attitudes and purchasing behavior. In a study of 240 Italian consumers, two distinct buyer profiles were identified. The first, nutrition-claim-oriented consumers, predominantly included women, individuals with higher nutrition knowledge, and families with young children, motivated by concern for household health. The second, health-claim-oriented consumers, consisted mainly of older individuals with lower incomes and existing health problems, who perceived health claims as particularly relevant for prevention and well-being. These findings suggest that health claims are especially effective among health-sensitive consumers and that their mode of presentation plays a crucial role in building trust and purchasing intention [112].

Packaging and labeling elements represent another important perceptual factor, shaping both product image and the credibility of health-related claims. Oliveira et al. [113] showed that consumers rarely analyze health claims in detail; instead, their perceptions are often driven by visual cues, such as color, symbols, or layout, rather than textual content [113]. A literature review by Ballco and Gracia [114] confirmed that purchasing behavior is influenced both by internal attributes (e.g., taste, composition) and external ones (e.g., brand, color, price, and communication content). Among individual-level factors, nutrition knowledge and health motivation are particularly important, while among external factors, health claims function as a signal of trust [114]. Overall, these findings indicate that labeling and message design play a crucial role in shaping perceived credibility, with visual elements often being more influential than detailed information [114].

Perceived naturalness is one of the most important criteria in choosing functional foods. Hosni et al. [9] demonstrated that authenticity and purity were the strongest predictors of purchase decisions, outweighing economic considerations. Similar conclusions were reached by Boccia et al. [100], who analyzed willingness to pay for natural foods among 2166 Italian consumers. Respondents were willing to pay more for products derived from natural production processes, with brand and transparency of origin also playing significant roles [100]. At the same time, the importance of health-related aspects in consumer decision-making continues to grow [115]. Awareness of healthy eating as a component of prevention and health maintenance is rising [116]. As a result, food quality is often perceived as equally important as price, and many consumers are willing to incur higher costs in exchange for quality and expected health benefits [24,25].

In the study by Hosni et al. [9] (*n* = 300, Greece), two consumer segments were identified using cluster analysis. The first group, price-concerned consumers (39%), was primarily guided by economic factors such as price, discounts, packaging, and exhibited low health awareness and limited willingness to pay more. The second group, health-concerned consumers (61%), included individuals with high health awareness who valued naturalness, purity, and nutritional quality. These consumers more frequently read labels, actively sought nutritional information, and were willing to pay 20–50% more for health-promoting products. This segment consisted mainly of individuals aged 25–34 with higher education and income levels. The results confirm the existence of two distinct market segments: one driven by economic considerations and another by health-oriented motivations [9].

Kraus [101], in a study of 200 Polish consumers, identified key attributes influencing purchasing decisions. The most important quality-related attributes included safety, naturalness, and perceived healthiness. The most valued health benefits were immunity support, reduction of cancer and cardiovascular risk, and weight maintenance. The most frequently indicated functional ingredients were vitamins, minerals, omega-3 fatty acids, and dietary fiber, while preferred carriers included bread, dairy products, cereal-based foods, fruit–vegetable products, and meat products. Both functional (e.g., health improvement) and psychological (e.g., conscious choice, health promotion) motivations were identified. The most frequently declared terminal values were good health, longevity, and health security [101]. These findings highlight that functional food consumption is strongly rooted in health-related motivations and broader life values.

Nazzaro et al. [117], Bock and Meyerding [118], Michałowska [119], Badshah et al. [120], Du and Duan [121], and Li and Li [122] emphasize the growing importance of external product attributes such as brand, packaging material, and labeling. Eco-friendly packaging design (e.g., color, biodegradable material, minimalist layout) significantly enhances perceptions of responsibility and trustworthiness. Green color, simple labels, and natural textures are commonly associated with authenticity and healthfulness, particularly among environmentally conscious consumers.

Price plays a dual role in the context of functional foods. It may act as a barrier to purchase, but it can also increase perceived healthiness and strengthen purchasing intention [5,123,124,125]. Huang et al. [23] showed that high price reduces purchase intention; however, this effect weakens among consumers with high health consciousness. Among individuals with low food neophobia, higher price may even be perceived as a signal of superior quality [23]. Müller-Pérez et al. [24], analyzing the behavior of 703 Mexican consumers, found that willingness to pay was a stronger predictor of purchase than price perception itself. Similarly, Katt and Meixner [126] demonstrated that health and environmental awareness can outweigh financial constraints, with consumers willing to pay more for products perceived as healthier and more sustainable.

In the study by Szczepańska et al. [102], knowledge of the term “functional food” in Poland remained limited, with only 42.5% of respondents recognizing it. Product composition and a healthy lifestyle were the main divers of purchase, while price remained the primary barrier. These findings suggest that although current consumption is relatively low, there is significant growth potential as consumer knowledge and awareness increase [102].

Perceptual and product-related factors determine how consumers evaluate the credibility and value of functional foods. Key elements include the congruence between the functional ingredient and carrier, sensory attributes, perceived naturalness, labeling clarity, and the balance between price and quality. Overall, consumers expect functional foods to combine health effectiveness with sensory appeal, transparency, and alignment with their environmental and aesthetic values.

### 4.4. Socio-Demographic and Segmentation Determinants

Age is one of the key predictors of acceptance of functional foods. In the study by Szakos et al. [3] conducted on a sample of 1002 Hungarian consumers, older individuals more frequently chose products enriched with vitamins, minerals, protein, and fiber. They perceived these products as part of disease prevention. Thus, the importance of functionality and safety increases with age, indicating a link between population aging and demand for foods supporting healthy aging. Similar results were reported by Corso et al. [104] among Brazilian consumers. Acceptance of functional foods increased with age, education, and income, while the main motivations were disease prevention and maintaining good physical condition.

In the study by Velli et al. [127], conducted in Canada among 200 individuals aged over 60, 93% of respondents declared consuming functional foods. Nutrition knowledge (85.5%) was identified as the strongest factor supporting consumption. More than 90% of respondents were familiar with health claims, and individuals with higher education were significantly more capable of interpreting them correctly. These findings suggest that both nutrition awareness and the need for health-promoting behavior increase with age. Firoozzare et al. [2,128] also reported that nutrition knowledge and awareness grow with age. Ballco and Gracia [114] and Bimbo et al. [129] confirmed that older individuals show greater willingness to choose foods perceived as healthier compared to younger consumers. In contrast, Vorage et al. [26], analyzing young adults aged 17–29 (*n* = 370, Australia), showed that positive attitudes toward functional foods were linked to naturalness and weight control motivations. However, actual consumption depended on life circumstances and level of health interest. These findings indicate that among young adults, functional food choices are often situational and lifestyle-driven rather than preventive.

Numerous studies confirm that women consume functional foods more frequently than men and exhibit more positive attitudes toward this category [36,130,131,132]. This may result from their greater involvement in food purchasing and meal preparation, as well as higher concern for health and appearance. The presence of children in the household is also an important motivating factor, as parents aim to provide a nutritious diet and reduce health risks [130]. As a result, functional foods are often perceived as an investment in family health.

Socioeconomic status also plays a significant role in functional food consumption. Individuals with higher education and income are more likely to be familiar with the concept of functional foods and show greater willingness to pay higher prices [104,133,134,135]. The study by Firoozzare et al. [2], conducted among 359 households in Iran, showed that higher age, income, nutrition knowledge, and health awareness significantly increased the likelihood of purchasing health-promoting foods. Conversely, larger household size reduces this likelihood, indicating that economic constraints remain an important barrier.

Plasek et al. [105] confirmed that higher education increases the likelihood of choosing immunity-supporting products. Individuals older than 36 years more frequently choose functional foods aimed at cardiovascular and digestive prevention. This suggests that consumer segments differ according to life stage and health orientation.

In the study by Horská et al. [103], conducted among 1138 Slovak consumers, three market segments were identified: (1) skeptics (52%)—characterized by low consumption and concerns about risk, (2) less interested (35%)—purchasing functional foods occasionally, often driven by trends or temporary interest, (3) enthusiasts (12%)—regularly consuming functional foods and convinced of their health benefits. Demographic analysis showed that enthusiasts were mainly younger, better educated, and professionally active, whereas skeptics were older and less nutrition-aware consumers [103].

Socio-demographic and segmentation factors shape purchasing behavior indirectly, primarily through their influence on knowledge, health awareness, and motivations. The findings are consistent in highlighting the growing importance of age, education, and income as factors supporting the acceptance of functional foods. At the same time, significant differences emerge between consumer segments. Older individuals tend to perceive functional foods as part of disease prevention, whereas younger consumers are more likely to treat them as elements of lifestyle and identity. Similarly, higher levels of education and income increase willingness to purchase, but are also associated with higher expectations regarding product quality and credibility. Consumer segmentation further reveals that the functional food market is highly heterogeneous—from skeptics to enthusiasts—indicating the need to tailor communication and marketing strategies to different target groups. Overall, these findings suggest that socio-demographic factors do not act as direct determinants of purchasing decisions but rather as moderating variables, strengthening or weakening the influence of other determinants depending on the social context and individual consumer profile.

## 5. Limitations, Implications and Future Research Directions

### 5.1. Limitations

This study has several limitations that should be considered when interpreting the findings. First, the review was based exclusively on publications indexed in the Scopus and Web of Science databases, which may have limited access to relevant studies available in other sources.

Second, only English-language publications were included, which may have resulted in the exclusion of valuable research published in other languages.

Third, the selection criteria focused primarily on empirical studies addressing consumer behavior. As a result, not all subtopics related to the functional food market are equally represented in the literature.

Fourth, certain areas—such as the role of new technologies, e-commerce, and personalized nutrition in shaping purchasing decisions—remain relatively underexplored.

Finally, due to the heterogeneity of the analyzed studies in terms of methods, samples, and cultural contexts, the conclusions presented in this paper are synthetic and interpretative rather than meta-analytical.

### 5.2. Theoretical Implications

The conducted review provides several important theoretical contributions to research on consumer behavior in the functional food market. First, the classification of determinants into four groups—health- and trust-related, cognitive and psychological, perceptual and product-related, and socio-demographic and segmentation factors—offers a more integrated perspective on the mechanisms shaping purchasing decisions. It highlights that consumer behavior toward functional foods cannot be explained solely by traditional food-related motives such as price, taste, or availability. Instead, it requires the inclusion of additional dimensions related to perceived health effectiveness, credibility of health claims, and the symbolic meaning of products.

Second, the review demonstrates that the functional food market differs from other food market segments in the stronger role of trust, knowledge, and perceived health benefits. While purchasing decisions in traditional food markets are often driven by habit, price, and sensory attributes, functional food choices involve an additional evaluation of claim credibility, product naturalness, and the congruence between functional ingredients and product carriers.

Third, the findings support the view that consumer behavior in this market is multidimensional and best explained through the integration of different theoretical perspectives, such as the Theory of Planned Behavior, the Health Belief Model, and the concept of Customer Perceived Value. This suggests that no single theoretical framework fully captures purchasing decisions in this category and that an integrative approach is required.

### 5.3. Practical Implications

The findings also provide important practical implications for functional food producers, marketers, and policymakers. First, building consumer trust in health claims is crucial. This requires transparent, reliable, and scientifically grounded communication based on credible sources and consistent messaging.

Second, effectively communicating product naturalness and authenticity remains a key challenge. Consumers expect not only health benefits but also transparency in composition, simplicity of labeling, and alignment with their environmental and health-related values. In practice, this implies the need to design both products and marketing messages that emphasize natural origin and credibility.

Third, producers should aim to balance health benefits with sensory quality. Taste and consumption pleasure remain essential for product acceptance; therefore, functional food development must combine health functionality with high sensory appeal.

Finally, marketing strategies should be adapted to diverse consumer segments, taking into account differences in knowledge, health motivation, and socio-demographic characteristics. For policymakers, the development of clear and consistent regulatory frameworks for health claims is particularly important, as it can support consumer trust and the stable development of the functional food market.

### 5.4. Future Research Directions

The findings of this review indicate several directions for future research. First, comparative studies across different food market categories are needed to better understand how determinants of functional food consumption differ from those related to organic, traditional, or convenience foods.

Second, further cross-cultural and cross-regional research is required to examine how cultural norms, regulatory environments, and dietary traditions influence the perception of health claims, naturalness, and trust in functional foods.

Third, future studies should focus more strongly on the relationship between sensory quality and perceived health benefits, as existing research suggests that this trade-off is one of the key factors determining market acceptance.

Fourth, longitudinal and experimental studies are needed to better capture changes in consumer attitudes and behaviors over time and to assess the impact of specific marketing communication strategies on purchase intention and actual behavior.

Finally, future research should further develop segmentation analyses by incorporating emerging factors such as digital information sources, the growth of e-commerce, personalized nutrition, and the increasing importance of sustainable consumption.

## 6. Conclusions

The findings of this review provide structured answers to the research questions and contribute to a more integrated understanding of consumer behavior in the functional food market. In response to RQ1, the analysis identified four main groups of factors shaping consumer purchasing decisions: health- and trust-related factors, cognitive and psychological factors, perceptual and product-related factors, and socio-demographic and segmentation factors. These groups reflect complementary dimensions of decision-making and confirm that functional food choices are driven by both rational and emotional mechanisms. In relation to RQ2, it can be concluded that the aforementioned factors influence consumer attitudes, purchase intentions, and actual behavior through complex interactions. Health benefits and trust serve as fundamental drivers of acceptance, while cognitive and psychological factors—such as self-efficacy, social norms, and identity—shape attitudes and intentions. At the same time, perceptual and product-related attributes, particularly taste, naturalness, and product format, are decisive for actual purchasing behavior. Socio-demographic variables do not directly determine choices but moderate the strength and direction of other determinants. Regarding RQ3, the review points to several key implications. From a theoretical perspective, the need for integrated approaches combining multiple frameworks (e.g., TPB, HBM, and perceived value theory) is crucial. From a practical perspective, the importance of building consumer trust, ensuring product transparency, and balancing health benefits with sensory quality is significant. Marketing strategies must also be tailored to diverse consumer segments. From a research perspective, further cross-cultural, longitudinal, and experimental studies are necessary, as well as deeper exploration of emerging factors such as digitalization, personalization, and sustainable consumption. In summary, consumer behavior in the functional food market is multidimensional and context-dependent. Key factors driving market growth include growing health awareness and the willingness to pay for credible and natural products, while limited knowledge and skepticism about health claims remain the main barriers.

## Figures and Tables

**Table 1:** Selected bioactive food components and their potential health effects.

Bioactive Component	Main Health Effects	Examples of Food Sources	References
Dietary fiber	Supports weight management and digestive function; reduces the risk of chronic diseases, including colorectal cancer; regulates blood cholesterol and glucose levels; exhibits anti-inflammatory and anticancer properties.	Fruits, vegetables, whole grains, legumes	[46,47,48,49]
Probiotics	Strengthen gut microbiota and immune function; support microbial balance in the intestines.	Yogurts, kefir, aged cheeses, fermented dairy products	[50,51,52,53,54]
Prebiotics	Stimulate the growth of beneficial gut bacteria; support digestion and immunity; may reduce the risk of inflammatory bowel diseases and cancers.	Onion, chicory, asparagus, garlic, bananas, honey, milk, wheat bran, oats, legumes	[51,52,55,56,57]
Omega-3 fatty acids (EPA, DHA, ALA)	Essential components of cell membranes; exhibit anti-inflammatory and cardioprotective effects; support treatment of inflammatory diseases and reduce triglyceride levels.	Fatty marine fish, fish oil, flaxseed, walnuts, algae	[57,58,59,60,61]
Flavonoids (catechins, quercetin, kaempferol)	Strong antioxidant and anti-inflammatory effects; support cardiovascular health; exhibit anticancer, antiviral, and antidiabetic properties.	Green tea, kale, onion, citrus fruits, dark chocolate, berries	[55,62,63,64,65,66,67,68]
Resveratrol	Exhibits antioxidant, anti-inflammatory, and anticancer effects; provides cardioprotective and anti-aging benefits.	Red grapes, red wine, berries	[69,70,71]
Lignans	Show anticancer and cardioprotective effects; help alleviate menopausal symptoms; may prevent osteoporosis and inflammatory diseases.	Flaxseed, cereals and cereal products	[72,73,74]
Tannins	Possess antioxidant, anti-inflammatory, antiviral, and antibacterial properties; support cardiovascular and gut health; may have prebiotic effects.	Legumes, nuts, red wine, pomegranates	[75,76,77,78]
Carotenoids (beta-carotene, lutein, zeaxanthin, lycopene)	Act as antioxidants; support eye health and immunity; reduce the risk of cancer and noncommunicable diseases.	Carrots, sweet potatoes, bell peppers, spinach, tomatoes, pink grapefruit, apricots, watermelon	[55,79,80,81]

**Table 2:** Conceptual Criteria Describing Functional Foods.

Criterion	Description
Health-promoting effects	Functional foods are characterized by scientifically verified properties that support specific physiological functions or help reduce the risk of selected chronic diseases and health disorders.
Product characteristics	Such foods may exist in traditional or modified forms, enriched with beneficial ingredients or reformulated to remove substances potentially harmful to health.
Nutritional and functional value	These products provide additional physiological benefits that go beyond basic nutrition, achieved through their composition, bioactive compounds, or technological processing.
Dietary applicability	Functional foods should be suitable for regular inclusion in everyday diets, aligning with established eating habits, as well as cultural and geographical practices.

Source: authors’ own elaboration based on [18,19].

**Table 3:** Determinants of consumer attitudes and behaviors toward functional foods—overview of international studies.

Country	Sample (*n*)	Analyzed Factors (Determinants)	Key Findings/Impact on Purchasing Behavior	References
Germany, China	502, 443	Health motivation, trust in the food industry, food neophobia, cultural factors	Chinese consumers showed significantly higher willingness to purchase functional foods. In both countries, higher health motivation and greater trust in the food industry increased willingness to buy functional products. Food neophobia negatively affected acceptance among Chinese consumers but not among German respondents. Findings highlight the role of cultural context in functional food acceptance.	[93]
New Zealand (Chinese-origin consumers)	193	Health concerns (mobility-related diseases), health motivation, trust in brand and manufacturer, advertising, willingness to pay (WTP)	Higher concern about mobility-related diseases and stronger health motivation increased willingness to purchase preventive functional foods. Trust in brand, country of origin, and authority-based advertising (e.g., scientists, public figures) positively influenced decisions. Most participants stated they would not pay more than 40% extra for additional health benefits.	[21]
China	1144	Trust in the food system, health consciousness, price, food neophobia	Trust and health consciousness positively influenced attitudes and purchase intentions. Price had a negative effect, although mitigated among highly health-conscious consumers. Neophobia did not directly affect purchase intention but moderated relationships: low-neophobia consumers viewed higher prices as financial sacrifice or a signal of quality.	[23]
Sri Lanka	280	Customer knowledge, necessity, safety, confidence, perceived rewards	Knowledge, perceived necessity, and safety negatively affected attitudes toward functional foods, whereas trust and perceived rewards acted positively. The strongest predictor of positive attitudes was the perceived health benefit (“reward”) from consumption.	[94]
Lithuania	900	Social and psychological motives: demonstrative consumption, self-control, influence of social norms	Demonstrative consumption and susceptibility to social norms positively affected perceived attractiveness and distinctiveness of functional foods, whereas self-control motivation had a negative effect. Results confirm the relevance of hedonic and social factors in functional food choices.	[27]
Norway	810	Factors influencing attitudes and intentions toward functional foods	The strongest predictor of intention to consume functional foods was self-efficacy—confidence in one’s ability to consume them regularly. Utilitarian values shaped attitudes positively, while hedonic values had a negative effect.	[95]
Belgium	215	Health beliefs, knowledge and awareness of functional foods, family health status, age	Acceptance increased with stronger belief in health benefits and presence of a chronically ill family member. High self-reported knowledge reduced acceptance, although this effect diminished with age. Cognitive and health-related factors were stronger determinants than demographics.	[96]
Finland	1158	Perceived rewards, trust, perceived necessity, perception of functional foods as medicine, nutritional risk, role in healthy diet, healthiness vs. taste	The strongest predictor of willingness to consume functional foods was perceived reward—the subjective sense of health benefit. Trust and low perceived risk also strengthened positive attitudes.	[22]
Hungary	1016	Expected taste, product familiarity, health image of the carrier, perceived fit of health effects	Expected taste and product familiarity were the strongest determinants. Perceived fit between carrier and functional ingredient had a smaller but significant positive impact (about +30%), especially among women and consumers with middle education and income.	[97]
China	1144	Food carrier, health benefits, trust in information channels, education	The food carrier was a stronger predictor of perceived attractiveness and purchase intention than health benefits alone. Benefits were evaluated more positively when aligned with an appealing carrier (e.g., yogurt, beverage). Products enhancing natural immunity were most preferred. Lower-educated consumers showed less willingness to purchase. While interpersonal communication was most trusted, trust in mass media more strongly predicted purchase intention.	[98]
Greece	300	Product characteristics (naturalness, nutritional value, economic factors)	Interest in functional foods was strongly linked to perceived naturalness and purity, and to a lesser extent to nutritional value. Economic factors (price, discounts, packaging) did not affect interest. Findings confirm naturalness as a key determinant of acceptance.	[9]
Australia	149	Food carriers and health claims; perceived attractiveness, credibility, novelty; experiment with 30 product concepts	Purchase intention was driven independently by attractiveness, credibility, and novelty, with attractiveness showing the strongest effect. The model explained 56% of variation in intention to try. The carrier was a stronger predictor than the health claim.	[99]
Italy	2166	Willingness to pay for functional products with attributes (brand, price, production method/naturalness)	Willingness to pay depended primarily on the naturalness of the production process, and also on brand and price. Naturalness was the key selection criterion.	[100]
Poland	200	Quality attributes, health properties, functional ingredients, carriers, motives and values	Key attributes included safety, naturalness, and perceived healthiness. Most desired health effects were immunity support, cancer and cardiovascular risk reduction. Preferred carriers included bread, dairy, cereals. Main motives: health improvement, body care, conscious choice, longevity.	[101]
Poland	301	Knowledge of functional foods, consumption drivers, product preferences, gender differences	Main drivers were healthy lifestyle (56.2%) and product composition (54.7%). Composition and price were top selection criteria. Overall knowledge and consumption were low, with no significant gender differences.	[102]
Hungary	1002	Age, attitudes toward food functionality	Acceptance increased with age, especially for products rich in vitamins, protein, fiber, and those lower in salt and sugar.	[3]
Slovakia	1138	Attitudes and beliefs, consumer segmentation	Three segments identified: skeptics (52%), less interested (35%), and enthusiasts (12%). Differences in perceived benefits and risks strongly determined consumption frequency.	[103]
Australia	370	Demographics and motives (naturalness, health, weight control, living situation)	Positive attitudes were driven by naturalness and weight control. Actual consumption depended on living situation, health motivation, and product naturalness. Logistic regression explained 14–18% of variance in attitudes and consumption.	[26]
Brazil	270	Age, education, income, health beliefs, sensory quality	Acceptance increased with age, education, income, and health knowledge. Price was not a significant predictor. Sensory quality was more important than price.	[104]
Hungary	1027	Age, education, type of illness, preventive motivation	Consumers most often chose functional foods to prevent digestive issues, weakened immunity, and high cholesterol. Higher education increased likelihood of choosing immunity-supporting products, while age >36 predicted preferences for cardiovascular and digestive prevention.	[105]

## Data Availability

This study is based solely on previously published data. All data sources are cited within the manuscript. No new data were created.
